# Tetrandrine ameliorates cognitive deficits and mitigates tau aggregation in cell and animal models of tauopathies

**DOI:** 10.1186/s12929-022-00871-6

**Published:** 2022-10-22

**Authors:** Benjamin Chun-Kit Tong, Alexis Shiying Huang, Aston Jiaxi Wu, Ashok Iyaswamy, Olivia Ka-Yi Ho, Anna Hau-Yee Kong, Sravan Gopalkrishnashetty Sreenivasmurthy, Zhou Zhu, Chengfu Su, Jia Liu, Juxian Song, Min Li, King-Ho Cheung

**Affiliations:** 1 School of Chinese Medicine and Mr. and Mrs. Ko Chi Ming Centre for Parkinson’s Disease Research, Hong Kong Baptist University, 7 Baptist University Road, Kowloon Tong, Kowloon, Hong Kong, SAR China; 2 Medical College of Acupuncture-Moxibustion and Rehabilitation, Guangzhou University of Chinese Medicine, Guangzhou, China

**Keywords:** Tauopathy, Tetrandrine, Lysosome, Calcium dysregulation, Two-pore channel 2, Autophagy

## Abstract

**Background:**

Tauopathies are neurodegenerative diseases that are associated with the pathological accumulation of tau-containing tangles in the brain. Tauopathy can impair cognitive and motor functions and has been observed in Alzheimer’s disease (AD) and frontotemporal dementia (FTD). The aetiology of tauopathy remains mysterious; however, recent studies suggest that the autophagic-endolysosomal function plays an essential role in the degradation and transmission of pathological tau. We previously demonstrated that tetrandrine could ameliorate memory functions and clear amyloid plaques in transgenic AD mice by restoring autophagic-endolysosomal function. However, the efficacy of tetrandrine and the associated therapeutic mechanism in tauopathies have not been evaluated and elucidated.

**Methods:**

Novel object recognition, fear conditioning and electrophysiology were used to evaluate the effects of tetrandrine on memory functions in transgenic tau mice. Western blotting and immunofluorescence staining were employed to determine the effect of tetrandrine on autophagy and tau clearance in vivo. Calcium (Ca^2+^) imaging and flow cytometry were used to delineate the role of pathological tau and tetrandrine in lysosomal Ca^2+^ and pH homeostasis. Biochemical BiFC fluorescence, Western blotting and immunofluorescence staining were used to evaluate degradation of hyperphosphorylated tau in vitro, whereas coculture of brain slices with isolated microglia was used to evaluate tau clearance ex vivo.

**Results:**

We observed that tetrandrine treatment mitigated tau tangle development and corrected memory impairment in Thy1-hTau.P301S transgenic mice. Mechanistically, we showed that mutant tau expression disrupts lysosome pH by increasing two-pore channel 2 (TPC2)-mediated Ca^2+^ release, thereby contributing to lysosome alkalinization. Tetrandrine inhibits TPC2, thereby restoring the lysosomal pH, promotes tau degradation via autophagy, and ameliorates tau aggregation. Furthermore, in an ex vivo assay, we demonstrated that tetrandrine treatment promotes pathological tau clearance by microglia.

**Conclusions:**

Together, these findings suggest that pathological tau disturbs endolysosomal homeostasis to impair tau clearance. This impairment results in a vicious cycle that accelerates disease pathogenesis. The success of tetrandrine in reducing tau aggregation suggests first, that tetrandrine could be an effective drug for tauopathies and second, that rescuing lysosomal Ca^2+^ homeostasis, thereby restoring ALP function, could be an effective general strategy for the development of novel therapies for tauopathies.

**Supplementary Information:**

The online version contains supplementary material available at 10.1186/s12929-022-00871-6.

## Background

Alzheimer’s disease (AD) is a chronic neurodegenerative disorder that affects more than 36 million people worldwide [1]. Most of the current FDA-approved treatments barely relieve AD symptoms, much less address the causes; effective therapeutic approaches are therefore urgently needed. The pathological hallmarks of AD are the aggregation of two insoluble proteins, amyloid β (Aβ) and tau, in the patient’s brain. The aggregation of each of these proteins is induced via different pathogenic mechanisms [2]. Mounting evidence suggests that the major underlying cause of the deposition of Aβ and tau is the body’s inability to clear them [3, 4]. Thus, the development of therapeutic approaches that promote the clearance of Aβ and tau aggregations appears to be a viable strategy for reducing their pathological effects [5–7].

Autophagy (i.e., macroautophagy), including the related autophagy-lysosomal pathway (ALP), is the primary mechanism by which aged organelles and aggregated or misfolded proteins are cleared [8]. The clearance of Aβ and hyperphosphorylated tau relies on degradation via the ALP; impairment of the ALP has been shown to exacerbate pathologies related to Aβ and tau [9]. Although ALP impairment is frequently observed in the brain in AD patients and transgenic AD models [10–14], the exact mechanisms leading to the impairment of ALP-mediated clearance of Aβ and tau are largely unknown. In spite of some controversy exists, studies have generally agreed that lysosomal alkalinization may be the cause of ALP impairment. Recently, we proposed a gain-of-function model to explain how lysosomal alkalinization may lead to impairment of ALP-mediated Aβ clearance in AD cellular and transgenic mouse models [14]. In trials with these models, we found that the activity of the lysosomal two-pore channel 2, TPC2, is increased in AD. The reduction of lysosomal calcium ions (Ca^2+^) drives the expulsion of hydrogen ions (H^+^) through the Ca^2+^/H^+^ exchanger (CAX), thus leading to lysosomal alkalinization and impairment of the ALP [14]. We also found that the TPC2 antagonist tetrandrine effectively corrects lysosomal pH, rescues ALP impairment, clears amyloid plaques and improves memory in transgenic AD mice [14]. This suggests that TPC2 is a potential therapeutic target and that its antagonist tetrandrine is a potential drug for AD treatment. The first step towards realizing this potential is to study the roles and effects of these factors in tau-induced pathologies (i.e., tauopathies).

Tauopathy is more strongly correlated with the cognitive decline and disease progression observed in AD than amyloid pathology [15]. Tau is a microtubule-associated protein. Excessive tau phosphorylation results in intraneuronal aggregations known as neurofibrillary tangles (NFTs) that impair axonal transport and functions [16]. In addition, it has been reported that hyperphosphorylated tau can block the trafficking of organelles and amyloid precursor protein-containing vesicles [17], synergistically accelerating disease pathogenesis [9]. Although tauopathy is commonly observed in AD, the associated genetic mutations have never been reported [18]. For this reason, an experiment in a pure AD tauopathy animal model is not feasible. Currently, the P301S/L mutation is commonly employed as a tauopathy model. The P301S/L tau mutation is identified in families with frontotemporal dementia (FTD) and parkinsonism [19] but not AD. Since tauopathies in AD are caused by the aggregation of the hyperphosphorylated protein [20], the P301S/L mutation can induce similar pathological features [18, 20]. Apart from that, the tau P301L mutation has been employed in the 3xTg-AD model [21]. Furthermore, the FTD tauopathy mouse model is commonly used, as it allows simpler evaluation of therapeutic approaches that target tau aggregation [22].

The present study evaluated and characterized the therapeutic effect of the TPC2 inhibitor tetrandrine as a lysosome-acidifying agent in tauopathy-based cell and animal models. Furthermore, we investigated the underlying molecular mechanism that disrupts pathologic tau clearance through endolysosomal maturation. Our in vivo study with Thy1-hTau.P301S mice shows that tetrandrine can mitigate tau-associated pathologies. This finding indicates that lysosomal acidity in ALP is impaired by pathological tau and that this effect can be corrected by tetrandrine. Mechanistically, our in vitro experiments confirmed that the expression of mutant tau-P301L protein increases TPC2 activity, causing lysosome alkalinization and ALP impairment. By inhibiting TPC2 overactivity, tetrandrine restores ALP impairment, reduces tau deposition, and mitigates associated tauopathies. Our study also demonstrated that tetrandrine enhances microglial clearance of tau NFTs, leading to reduced neuroinflammation and improved memory functions in a dose-dependent manner. In conclusion, reacidifying lysosomes with tetrandrine could be a viable and effective therapeutic approach for tauopathy intervention.

## Materials and methods

### Animal model

In this study, Thy1-hTau.P301S mice were employed as a tau mouse model, and C57BL6/J (C57) mice were employed as controls. The Thy1-hTau.P301S founder mice had a CBA x C57BL6 mixed genetic background. These mice were back-crossed with C57 mice to establish a homozygous Thy1-hTau.P301S line [23]. C57 mice were therefore employed as a wild-type reference for Thy1-hTau.P301S in other similar studies [24–26].

### Animal experiments

All animal protocols and procedures were performed in compliance with the recommendations of the NIH “Guide for the Care and Use of Laboratory Animals” and were approved by the Research Ethics Committee (REC) on the Use of Live Animals in Teaching and Research of Hong Kong Baptist University (REC/19–20/0199). C57 mice were obtained from the Jackson Laboratory (Bar Harbor, ME, USA). Homozygous human P301S tau transgenic Thy1-hTau.P301S mice were a generous gift from Dr. Michael Goedert [23]. They were housed in a pathogen-free facility under a 12-h light, 12-h dark cycle, with food and water provided ad libitum from birth. The mice were randomly divided into four equal groups according to the tetrandrine dose (0 mg/kg, 2.5 mg/kg, 5 mg/kg, and 10 mg/kg). Tetrandrine or saline was delivered by intraperitoneal injection every two days starting when the mice were two months old. Treatment efficacy was assayed when the mice reached four months of age. Behavioural experiments, contextual fear conditioning (CFC), novel object recognition (NOR), hippocampal long-term potentiation (LTP) measurement, immunohistochemistry for tau NFTs and Western blot analyses for hyperphosphorylated tau aggregates were performed.

### Contextual fear conditioning (CFC) and novel object recognition (NOR)

CFC and NOR were used for behavioural studies. Drug treatments were continued as scheduled throughout the test.

For CFC, contextual and cue tone-associated fear conditioning tests were used to evaluate the effect of tetrandrine on cognitive deficits. In brief, training was performed on Day 1. A mouse was placed in a specially designed sound-proof chamber and left for 2 min. After that, the mouse was stimulated with a 30 s tone at 70 dB followed by a foot shock (2 s at 1 mA). The whole process was repeated 3 times for each mouse. Cue tone-associated fear conditioning was conducted to assay nonhippocampal memory in a novel environment. Each mouse was stimulated by a cue tone without an electrical shock. Freezing behaviour indicated the recall of memory associated with shock. After the mouse rested for 2 h, CFC was conducted on Day 2 to assay hippocampus-associated memory functions using the same chamber setting without the cue tone or shock. The acquired data, including tones, shocks and freezing behaviour, were analysed using ANY-maze software (Stoelting Co., Wood Dale, IL, USA).

For NOR, a 2-object novel object recognition test was employed to evaluate recognition memory. In brief, each mouse was allowed to freely explore an empty arena one day before the test. On Day 1 of training, each mouse was exposed to the familiar arena with two identical objects placed equal distances from the centre. On Day 2, one of the objects was replaced with another object of the same size but a different shape and appearance. The coordinates of the locations of the mouse heads were traced to evaluate the time spent exploring each object. The recognition index (RI) was calculated by RI = T_N_/(T_N_ + T_F_), where T_N_ and T_F_ represent the time spent investigating the novel object and familiar object, respectively [27]. The recognition index percentage was calculated using ANY-maze software.

### Hippocampal long-term potentiation (LTP) recording

To investigate the functional effects of tetrandrine-mediated tau clearance in tau mice, the evoked field excitatory postsynaptic potential (fEPSP) of Schaffer collaterals was measured in the cerebral region between hippocampal CA1 and CA3. The theta burst-induced hippocampal long-term potentiation (LTP) was scored as a measure of neuronal plasticity. Freshly cut brain slices (350 µm) were prepared in ice-cold sucrose-substituted aCSF (artificial cerebrospinal fluid) using a vibratome (Campden Instruments, UK), recovered in oxygenated aCSF and incubated at 32 °C for 2 h. fEPSP was recorded by a MED-64 multielectrode array system (Alpha Med Scientific, Japan) with freshly oxygenated aCSF perfused in an open loop style. Five chains of theta bursts (TBS, 10 burst pulses at 100 Hz with 200-ms intervals for each pulse and 30-s intervals for each train) were delivered to stimulate LTP. The increase in fEPSP was monitored and assayed for 60 min after stimulation to ensure that the LTP event was stable.

### Immunohistochemistry

Paraformaldehyde-fixed brains were sectioned with a cryostat. For double immunostaining, AT8 and Iba-1 (microgliosis) or a GFAP (astrocytosis) combination were used to detect the colocalization of these antigens with tau tangles. Sections through each anatomic region of interest were imaged, and a threshold optical density was obtained that discriminated staining from the background. Images were analysed using ImageJ analysis (NIH) software.

### Cell culture

The human neuroblastoma cell line SH-SY5Y was cultured in Dulbecco’s modified Eagle’s medium (DMEM)/F-12 (Invitrogen) supplemented with 10% foetal bovine serum (Biosera, France) and 50 U of penicillin‒streptomycin mixture (Invitrogen) at 37 °C and 5% CO_2_. Primary microglia were prepared and separated according to the protocol described in [28]. They were cultured in DMEM supplemented with 10% foetal bovine serum and used after being cultured for 10 days in vitro (DIV). Unless otherwise specified, the duration of tetrandrine and bafilomycin A1 treatment in the in vitro experiment was 6 h.

### *Single-cell Ca*^*2*+^*imaging*

Single-cell fluorescence imaging was used to monitor intracellular Ca^2+^ signals as described in [14]. In brief, lysosomal Ca^2+^ content is assayed by changes in cytosolic Ca^2+^ after the addition of the lysosome osmolytic agent GPN (400 µM) to rupture lysosomes. Cytosolic Ca^2+^ was monitored by Fura2-AM (Invitrogen), and the ratios of emitted fluorescence were converted to Ca^2+^ concentration, [Ca^2+^]_i_, according to the Grynkiewicz equation, as previously described [29, 30]. In some experiments, changes in lysosomal Ca^2+^ were monitored using TPC2-GCaMP6m (Addgene; #80147) as described previously [31]. In brief, lysosomal TPC2 Ca^2+^ was discharged by the addition of NAADP-AM, and the changes in lysosomal Ca^2+^ were reflected by the changes in the TPC2-GCaMP6m fluorescence level [31]. ImageJ software with Micro-Manager was used for image capture and data analyses [32].

### Tau internalization and trafficking assay

To assay the internalization and trafficking of tau aggregates, insoluble tau aggregates isolated from the sarkosyl-insoluble fraction of tau-P301S mouse brain homogenates were prelabelled with FITC-AT8 antibody and incubated with microglial cultures. The abundance of internalized tau aggregates was then monitored by flow cytometry.

### Tau cleavage and clearance assays

The clearance of tau protein was studied as previously described [33, 34]. In brief, tau protein was tagged with EGFP or mTagBFP2 and cotransfected with RFP-LC3 into our in vitro cell models. The colocalization of mTagBFP2-tau or mTagBFP2-tau-P301L with RFP-LC3 was monitored by confocal microscopy.

### Ex vivo tau clearance assay

To investigate tau clearance by microglia in the brain, an ex vivo tau clearance assay was performed using an approach similar to that described by Luo et al. [35]. In brief, unfixed brain sections were prepared from tau-P301S mice using a cryostat and incubated with microglia at 37 °C for 24 h. The clearance of tau tangles was evaluated by quantitative immunohistochemistry.

### Western blot analysis of tau aggregates

Whole-brain lysate was homogenized and centrifuged at 110,000 × *g* for 30 min. The pellet was extracted in a high-salt buffer and further dissolved in 1% sarkosyl (high-salt buffer). Sarkosyl-soluble and Sarkosyl-insoluble fractions were subjected to SDS‒PAGE and probed with the following tau antibodies: HT7 (Invitrogen), AT-8 (Invitrogen), PHF-1 (a generous gift from Dr. Peter Davies) and MC-1 (a generous gift from Dr. Peter Davies). HT7 is used to detect human total tau (residues 159–163). AT8 and PHF-1 are used to detect phosphor-tau (AT8: Ser202, Thr205; PHF-1: Ser396, Ser404). MC-1 was used to detect misfolded tau (residues 312–322).

### Microsphere phagocytosis assay

Phagocytosis was assayed using fluorescent microspheres [36]. Cells were incubated with Sarkosyl-insoluble fractions from tau-P301S mouse brain homogenates with or without tetrandrine treatment. After treatment, fluorescent microspheres were added to the cells and incubated at 37 °C for 30 min. Free microspheres were removed by washing the cells twice with PBS. The phagocytosed microspheres were observed by confocal microscopy.

### Quantitative real-time PCR (RT‒PCR)

Total RNA from the mouse hippocampus was isolated with TRIzol reagent (Thermo Fisher Scientific, #15596018) according to the manufacturer’s instructions. First-strand cDNA was synthesized with 2 μg of total RNA using a High-Capacity cDNA Reverse Transcription Kit (Thermo Fisher Scientific, #4374966). Real-time PCR was performed using SYBR Premix Ex Taq with ROX reference dye (TaKaRa, RP420A) in a ViiA 7 Real-Time PCR system (Applied Biosystems). Relative expression was calculated using the ΔCT method and normalized to beta-actin. The following primers were used:

beta-actin: AGAGGGAAATCGTGCGTGAC, CAATAGTGATGACCTGGCCGT;

CREB1: CACAGACCACTGATGGACAGCA, AGGACGCCATAACAACTCCAGG;

CREB3: GGCTGATACTGACCGAGGAAGA, GAATCTTCCTCCGCACTCGTTTC;

CREB5: GCAAGGTCCAAACCTCAGCAAC, TGTCCGATGGTGCTCATGTTCC;

TPC1: CCCTGGAGTTACCTCGTGTTTC, GAATGCCGTGACCGAGAAATCG;

TPC2: CATCCACCTGTGTCTCTTCACC, GTGAGGTCAGTGCTTCTGGAAG;

NCX1: GAGAGCATTGGCATCATGG, ACCTCCAGCTTGGTGTGCTCG;

NCX2: GGAGCATCTTTGCCTATGTCTGG, TTGTCCGCCATCCAGGCAAACA;

NCX3: GGACCAGTTCATGGAAGCCATC, CACAGGCAAAGAGCACCTTCCA;

NHE6: GGAAAGTGTCCTCAATGACGCG, GAACATGGCTGTAACGTCAAAGG;

NHE7: CCTTCTTCCTCAACTTGGGCAG, CATACGATGCCGTGTCACGGAT; and

NHE9: TCTTGGAGTGCCTTCCTGTCTG, GGTCCTCAGTTTGGAATCCGATG.

### Data processing and analysis

All data were analysed by GraphPad Prism software. Unless specified otherwise, data are summarized as the means ± SEMs. Multiple comparisons between groups were performed by one-way ANOVA. Bonferroni’s test was used for post hoc comparison with the control group. *P* < 0.05 was considered statistically significant.

## Results

### Tetrandrine ameliorates tauopathies in an Thy1-hTau.P301S animal model

A pharmacokinetics study showed that the TPC2 inhibitor tetrandrine has good brain bioavailability and significantly mitigates amyloid pathologies in AD animals [14]; however, its effects on a tauopathy model have not been investigated. To study the in vivo effect of tetrandrine on tau-associated pathology, we employed Thy1-hTau.P301S mice as a tauopathy model [23].

**Fig. 1:**
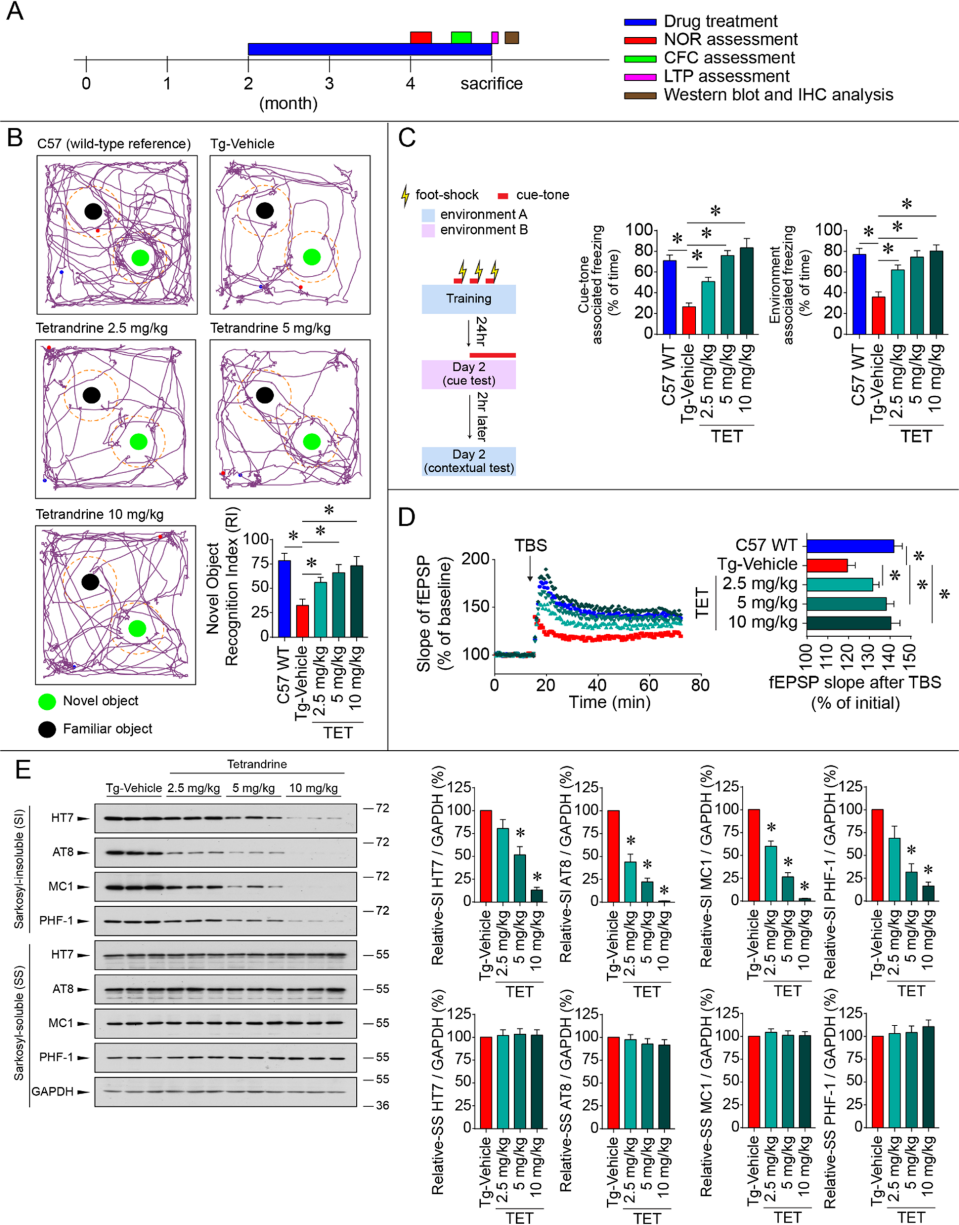
Tetrandrine ameliorates memory dysfunction and tau aggregation in tau mice. **A** A timeline diagram depicting the drug treatment and experimental plan of the study. Tau mice (Thy1-hTau.P301S, Tg) were treated with saline (Tg-vehicle), and different concentrations of tetrandrine (Tg-TET; 2.5 mg/kg, 5 mg/kg, and 10 mg/kg) via intraperitoneal (i.p.) injections every two days starting when the mice were 2 months old. When the mice reached 4 months old, they were subjected to behavioural and electrophysiological tests and Western blot and immunohistochemical analyses. **B** The novel object recognition test (NOR) was used to assay memory function. Mice were allowed to freely explore a familiar arena and were exposed to two identical objects (familiar object) prior to the experiment. Representative graphs show the exploration tracks of the mice after one of the objects was replaced by a new object (novel object). The familiar object and novel object are shown as black and green circles, respectively. The wild-type C57 mice spent more time in the exploration zone (shown as a dotted line) of the novel object than the Tg mice. Treatment with tetrandrine increased the exploration of novel objects by Tg mice in a dose-dependent manner. The bar chart depicts the recognition index (RI) calculated by the time the mice spent with each object. Data are summarized as the mean ± SEM from 8 mice in each group. **C** The contextual fear conditioning test (CFC) was used to assay learning and memory functions. The experiment was conducted as shown on the left. A mouse was placed in a specially designed sound-proof chamber and was stimulated by a foot shock coupled with a cue tone three times. The mice were then assayed in a novel environment with cue tones played after 3 min of exploration or in the same environment without cue tones to assay cue tone-associated and context-associated fear memory. Freezing behaviour represented the recall of memory associated with the electrical shocks. Bar charts depict the percentage of freezing in contextual and cued tests. Data are summarized as the mean ± SEM from 8 mice in each group. **D** Representative traces showing the electrophysiological fEPSP measurements evoked by 5 trains of theta burst stimulation (TBS) in brain slices isolated from C57 WT mice treated with different concentrations of tetrandrine (Tg-TET) or vehicle (Tg-Vehicle). The bar chart summarizes the fEPSP after TBS stimulation. Data are summarized as the mean ± SEM from 8 mice with 3 brain slices each from each group. **E** Western blot analyses showing the amount of phosphor-tau protein in Sarkosyl-soluble (SS) and Sarkosyl-insoluble (SI) fractions in wild-type C57 mice (C57 WT) or mice treated with different concentrations of tetrandrine (Tg-TET) or vehicle (Tg-Vehicle) probed with different tau antibodies. Bar charts depict the amount of tau protein normalized to GAPDH in the SS and SI fractions. Data are summarized as the mean ± SEM from 8 mice in each group. *Indicates *p* < 0.05 compared with the C57 WT group or tau mice treated with saline (Tg-Vehicle)

Thy1-hTau.P301S mice stably overexpress human mutant tau P301S in the brain and do not carry other AD-related mutations, such as mutations in presenilin or amyloid precursor protein. Thus, this model provides a clean genetic background to evaluate the therapeutic effect of tetrandrine on tauopathy. Since the pathology of this line starts to develop from 3 months of age [37], we treated the mice with tetrandrine from 2 to 4 months of age to evaluate whether tetrandrine treatment could prevent or reduce the NFTs, contextual fear memory deficits and LTP impairment that have been reported in these tau mice (Fig. 1A). Our results showed that Thy1-hTau.P301S mice have memory and learning deficits compared with wild-type C57BL6/J (C57) mice. In the NOR test, the transgenic tau mice (Tg-Vehicle) spent less time with the novel object than the wild-type C57 mice (Fig. 1B). Tetrandrine treatment increased the time spent with the novel object by Thy1-hTau.P301S mice in a dose-dependent manner, suggesting improvements in both memory and learning functions in the tau mice (Fig. 1B). For the CFC test, cue tone- and context-associated freezing behaviour was significantly decreased in Thy1-hTau.P301S mice compared with wild-type C57 mice. The reduction in freezing behaviour in tau mice was restored by tetrandrine in a dose-dependent manner (Fig. 1C). We also measured electrophysiological fEPSP in brain slices isolated from wild-type C57, tau mice and tau mice treated with tetrandrine. The results indicated that LTP was impaired in tau mice and was rescued by tetrandrine treatment in a dose-dependent manner (Fig D). To assess tau NFT development in Thy1-hTau.P301S mice, we performed Western blot analyses using several tau antibodies recognizing different epitopes of hyperphosphorylated tau or tangles, and the results showed that tetrandrine reduced insoluble phosphor-tau and insoluble total tau protein levels in a dose-dependent manner in tau mouse brain tissues (Fig. 1E), suggesting pathological tau aggregates are reduced after tetrandrine treatment.

**Fig. 2:**
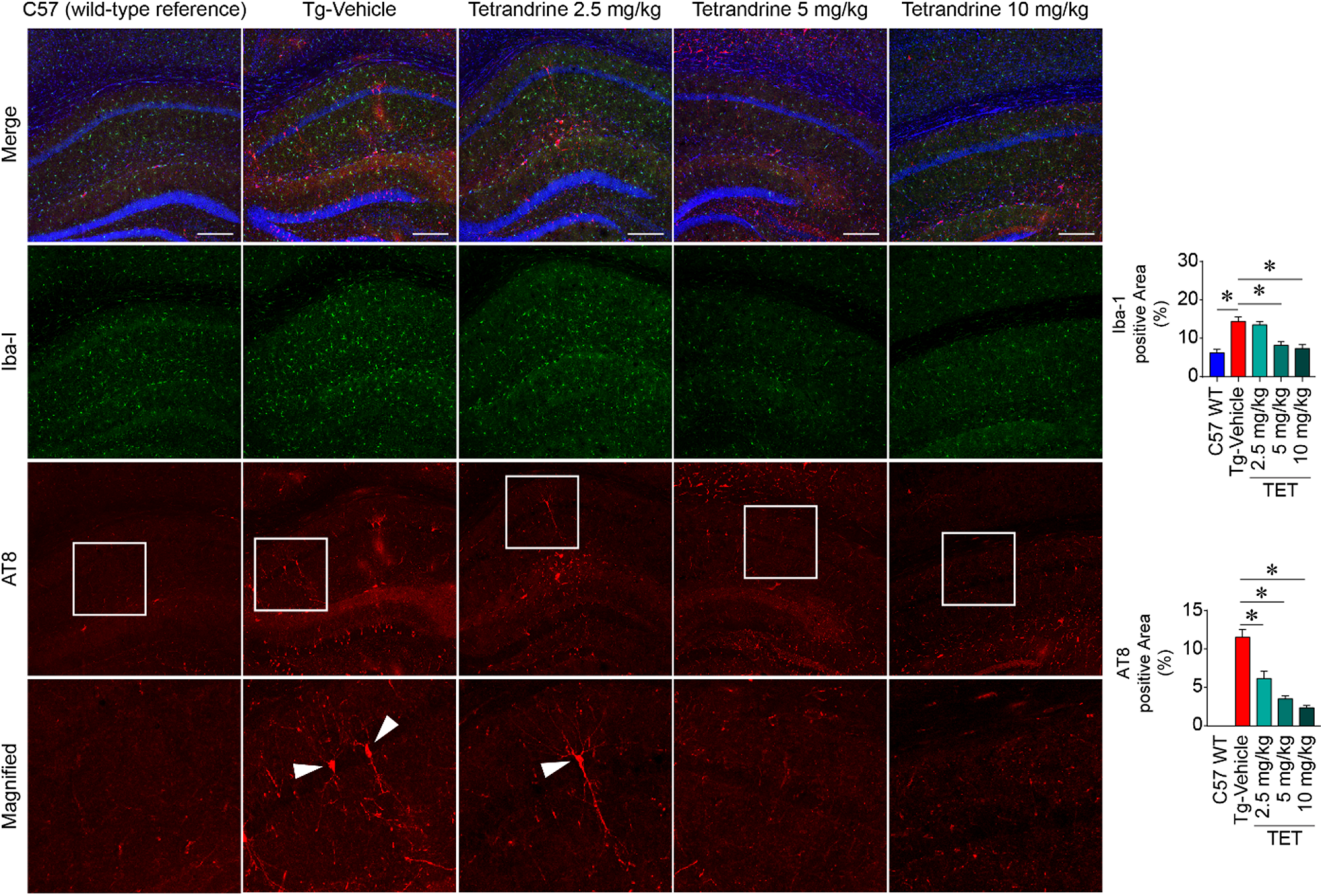
Tetrandrine reduces Iba-1 (gliosis) and AT8 (phosphorylated tau) signals in Thy1-hTau.P301S mice. Micrographs showing representative immunofluorescence staining of gliosis and phosphor-tau in hippocampal regions of control wild-type C57 (C57 WT) or Thy1-hTau.P301S mice treated with saline (Tg-Vehicle) or different concentrations of tetrandrine (TET; 2.5 mg/kg, 5 mg/kg, and 10 mg/kg, ip injection every two days from 2 months old to 4 months old). After the treatment, brain slices were immuno-probed with Iba-1 and AT8 antibodies to reveal gliosis and hyperphosphorylated tau. The bottom panel shows the magnified region in the white boxes in the AT8 immunostaining panels. The arrowheads of the magnified micrographs depict neurons with hyperphosphorylated tau. Quantifications of Iba1 and AT8 levels are shown in the bar charts. Data are summarized as the mean ± SEM from 8 mice, with 24 images analysed in each group. *Indicates *p* < 0.05 compared with C57 WT or vehicle-treated tau mice control

In addition to Western blot analyses, AT8/GFAP/Iba-1 immunohistochemistry was also used to evaluate neuroinflammation in brain cryosections from wild-type C57 and Thy1-hTau.P301S mice, and Thy1-hTau.P301S mice treated with different concentrations of tetrandrine. We observed substantial amounts of phosphor-tau proteins (AT8) in brain tissues of Thy1-hTau.P301S mice, and these protein accumulations were accompanied by increased expression of GFAP and Iba-1 (markers of astrocytosis and gliosis, respectively) compared with that in wild-type C57 mice (Fig. 2 and Additional file 1: Fig. S1). The increase in phosphor-tau protein and the accompanying astrocytosis and gliosis in tau mice were resolved in a dose-dependent manner by tetrandrine treatment (Fig. 2). Altogether, this in vivo study revealed that tetrandrine can reduce tau aggregation and alleviate tau-associated memory impairment in the Thy1-hTau.P301S animal model.

### Hyperphosphorylated tau enhances lysosomal TPC2 activity and impairs autophagy

**Fig. 3:**
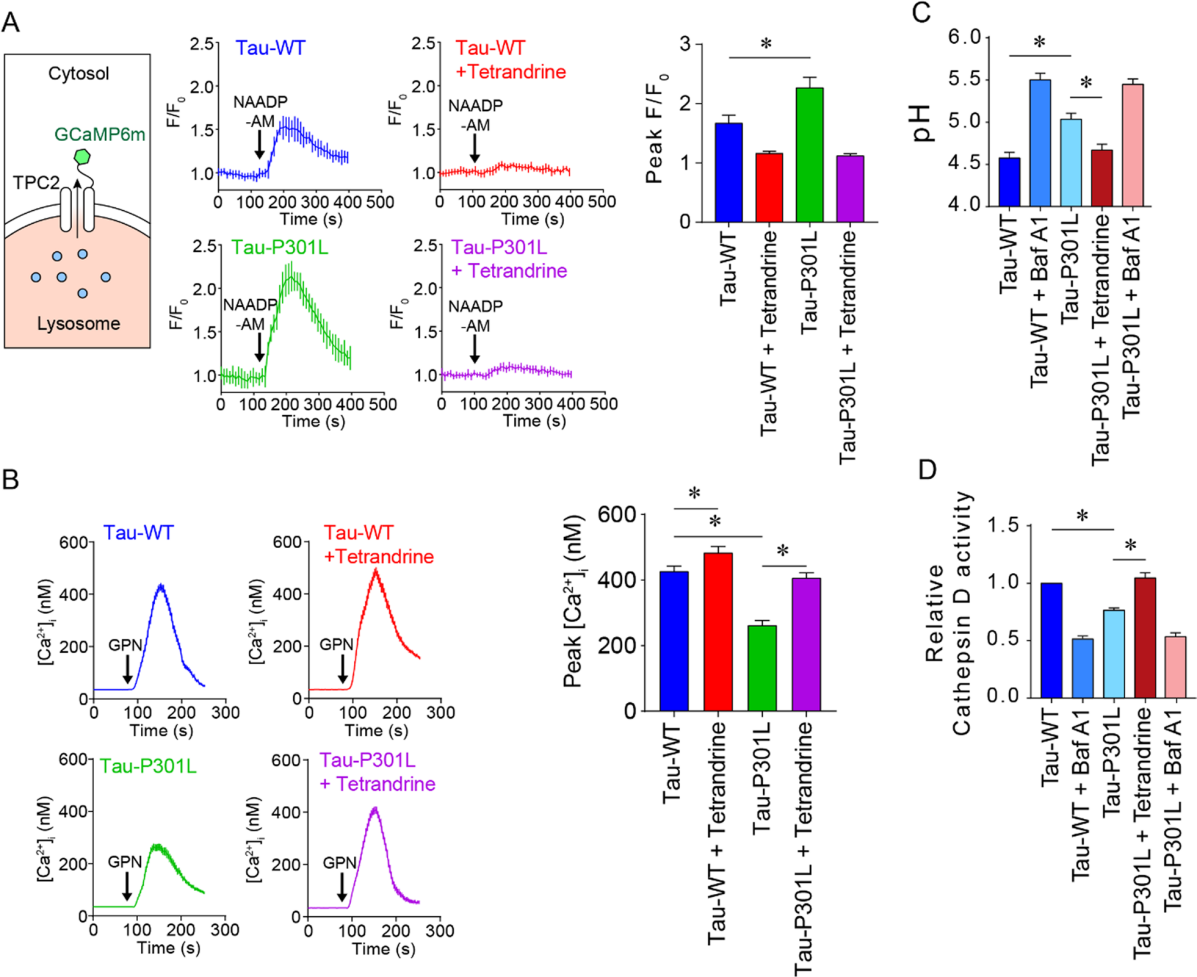
Pathological tau disrupts lysosomal Ca^2+^ and pH homeostasis by increasing TPC2 activity. **A** Left, a diagram depicts the topological representation of transfected GCaMP6m-TPC2 in the lysosome. Ca^2+^-sensitive GCaMP6 is located on the cytoplasmic side and can sense Ca^2+^ released from lysosomes. Middle, representative Ca^2+^ traces showing lysosome Ca^2+^ efflux measured by the expressed GCaMP6m-TPC2 in WT or mutant tau-expressing SH-SY5Y cells. The bar chart on the right shows the normalized peak fluorescence intensity (F/F_0_) of GCaMP6m-TPC2, summarized as the mean ± SEM from 3 individual experiments, with 90 cells analysed in each group. **B** Representative Ca^2+^ traces depict the changes in cytoplasmic Ca^2+^ content measured by the Ca^2+^ indicator Fura-2AM after the addition of GPN to SHSY5Y cells expressing Tau-WT or Tau-P301L with or without tetrandrine treatment. Each trace summarizes the changes in cytoplasmic Ca^2+^ in 90 cells from 3 experiments. The bar chart on the right shows the peaks of GPN-induced cytoplasmic Ca^2+^ changes. **C** Lysosomal pH measurements of SH-SY5Y cells expressing Tau-WT or Tau-P301L. The V-ATPase inhibitor bafilomycin A1 (BafA1) was used as a control. **D** Lysosomal cathepsin D (Cat D) activity measured in SH-SY5Y cells expressing Tau-WT or Tau-P301L. BafA1 was used as a control. Unless otherwise specified, all data are summarized as the mean ± SEM from at least 3 individual experiments; *Indicates *p* < 0.05

Tetrandrine is a potent TPC2 inhibitor. It has been shown to mitigate amyloidopathy by suppressing the TPC2-mediated lysosome alkalinization process [14]. Lysosomal alkalinization mediated by TPC2 is coupled with Ca^2+^/H^+^ exchanger (CAX) function. Although the identity of the CAX is not known in mammals, it is believed that CAX function exists and is mediated by the combined activities of sodium hydrogen exchangers (NHEs) and sodium calcium exchangers (NCXs) [38]. We found that the expression levels of NHEs and NCXs remained unchanged in Thy1-hTau.P301S tau mice (Additional file 1: Fig. S2). Instead, we observed upregulated TPC expression in Thy1-hTau.P301S tau mice (Additional file 1: Fig. S3), suggesting that lysosome acidity may be affected in tau mice. Together with the therapeutic effect of tetrandrine on Thy1-hTau.P301S tau mice, these findings suggest that autophagy‒lysosome pathway (ALP) impairment is also manifested in tauopathy. To investigate whether hyperphosphorylated tau impairs the ALP, we employed the SH-SY5Y human neuronal cell line, which stably expresses mutant tau-P301L, as a cellular model. Using NAADP-AM as a TPC2 agonist and TPC2-GCaMP6m as a lysosomal Ca^2+^ probe, we measured the changes in lysosomal Ca^2+^ content under the influence of tau protein phosphorylation. Stimulating the cells with NAADP-AM triggers Ca^2+^ release through lysosomal TPC2, which can be detected by the TPC2-GCaMP6m Ca^2+^ probe (Fig. 3A). In tau-P301L-expressing cells, the NAADP-elicited Ca^2+^ transient was significantly elevated compared with that in cells expressing wild-type tau (Fig. 3A). Using GPN (glycyl-L-phenylalanine-beta-naphthylamide, 400 µM) as an osmolytic agent, we observed a reduced lysosomal Ca^2+^ content in tau-P301L-expressing cells compared to tau-WT-expressing cells (Fig. 3B). These lysosome Ca^2+^ deficits in tau-P301L-expressing cells could be rescued by the TPC2 inhibitor tetrandrine and restored to a level comparable with that in tau-WT-expressing cells (Fig. 3A, B). These results suggest that lysosome Ca^2+^ homeostasis is disrupted by hyperphosphorylated tau protein. To verify whether the gain of function of TPC2 in mutant tau-expressing cells can promote lysosomal alkalinization, we measured lysosome pH using LysoSensor DND-160 [14]. Our results showed that tau-P301L-expressing cells had a slightly higher lysosomal pH (pH 5.0) than wild-type tau-expressing cells (pH 4.6), which was similar to the pH of Tau-WT-expressing cells treated with the V-ATPase inhibitor bafilomycin A1 (Baf A1, Fig. 3C). The increase in lysosomal pH in mutant tau-expressing cells could be reduced by tetrandrine (Fig. 3C), thereby reacidifying lysosomes. pH alterations may disrupt lysosomal degradation of tau aggregates, for example, via cathepsin D (CatD) [39]. To assess whether lysosomal alkalinization affected the activities of lysosomal enzymes during autophagy, we compared CatD activity in wild-type and mutant tau-expressing cells. Using BODIPY FL-conjugated Pepstatin A (Invitrogen) as a probe in a flow cytometry assay, we showed that CatD activity was significantly reduced in mutant tau-expressing cells, and this reduction in CatD activity could be restored to a level resembling that in tau-WT-expressing cells after tetrandrine treatment (Fig. 3D). Taken together, our cellular data suggest that ALP is impaired in mutant tau-expressing cells, that this impairment is related to disruptions in lysosomal Ca^2+^ and pH, and that tetrandrine can effectively rectify this ALP impairment.

### Tetrandrine restores lysosomal enzyme activities and ameliorates ALP-mediated tau degradation

**Fig. 4:**
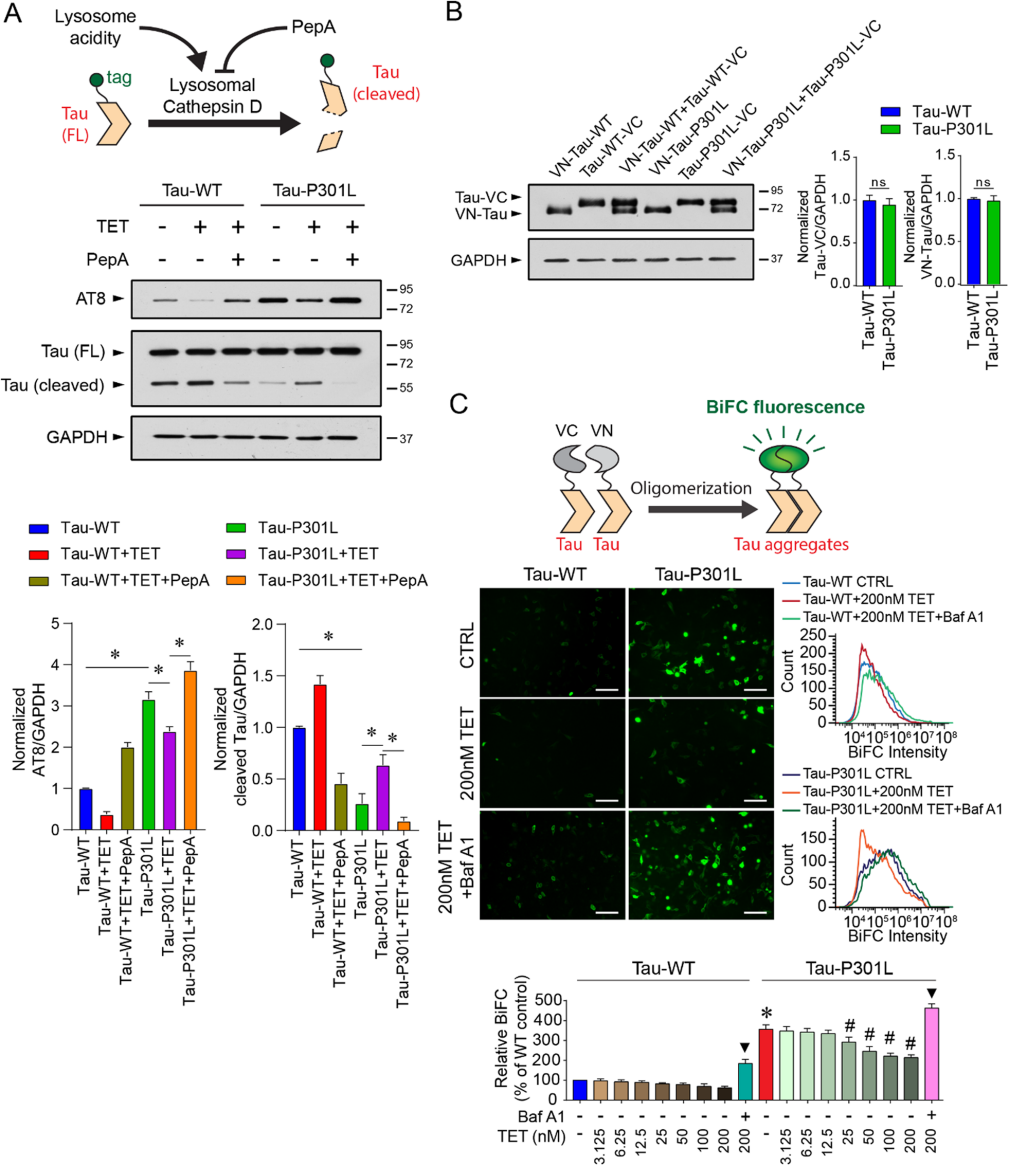
Tetrandrine ameliorates tau aggregation through endolysosomal degradation. **A** Top, schematic representation showing the manipulation of cathepsin D activity by tetrandrine or pepstatin A (Pep A) on the aggregation of tagged-tau ion SH-SY5Y cells. Middle, Western blot analyses of tau aggregation under the influence of tetrandrine and pepstatin A in Tau-WT- or Tau-301L-expressing SH-SY5Y cells. Cathepsin D activity was modulated by the addition of tetrandrine (TET) or Pep A. Bar charts depict the protein amount of cleaved and uncleaved tau normalized to GAPDH. Data are summarized as the mean ± SEM from 3 individual experiments; *Indicates *p* < 0.05. **B** Western blot analysis of the expression levels of VN and VC fragments of biomolecular fluorescence complementation (BiFC) in Tau-WT- or Tau-P301L-expressing cells. The bar chart depicts the protein levels of VN-tagged and VC-tagged tau when they were coexpressed. Expression of Tau-WT or Tau-P301L did not affect the expression of VN- and VC-fragments in cells. Data are summarized as the mean ± SEM from 3 individual experiments. n.s. denotes no statistical significance. **C** Top, a schematic showing the formation of BiFC by the expressed VN- and VC-fragments. Middle, representative micrographs showing the fluorescence levels of BiFC in Tau-WT- or Tau-P301L-expressing cells under the influence of tetrandrine with or without BafA1 cotreatment. Bottom, representative flow cytometry data depict the effect of tetrandrine (TET) or Baf A1 on tau aggregation. The bar chart depicts data summarized as the mean ± SEM from 3 individual experiments; * and ^#^Indicate *p* < 0.05 compared to the WT and untreated mutant tau groups, respectively. ▼ indicates *p* < 0.05 compared to the 200 nM tetrandrine-treated groups

To investigate whether tetrandrine promotes enzymatic degradation of hyperphosphorylated tau through the ALP, human neuroblastoma SH-SY5Y cells were transfected with GFP-tagged mutant tau-P301L or wild-type tau. We manipulated CatD activity by acidifying lysosomes with tetrandrine or inhibiting CatD with pepstatin A [39]. We then measured tau degradation by Western blot analysis using anti-GFP antibodies (Fig. 4A). Mutant tau expressed in SH-SY5Y cells provoked tau aggregation, as shown by the increase in the intensity of the AT8 immunoreactive band (Fig. 4A). In addition, we observed that the amount of cleaved tau fragment was significantly reduced in mutant tau-expressing cells compared to cells expressing WT tau (Fig. 4A). Treating the cells with tetrandrine restored tau cleavage to a level comparable to that observed in WT tau-expressing cells. Tau cleavage was diminished when mutant tau-expressing cells were cotreated with tetrandrine and pepstatin A, indicating that tau cleavage is a CatD-dependent process (Fig. 4A).

As some studies have suggested that tau aggregation can be relieved by autophagic degradation [40], we further evaluated the effect of tetrandrine on tau aggregation using a bimolecular fluorescence complementation (BiFC) assay as a tau oligomerization reporter [41]. In the assay, we tagged WT or mutant tau with C-terminal (VC) and N-terminal (VN) fragments of a yellow fluorescent protein and coexpressed these fragments in SH-SY5Y cells. We found that the protein abundance of VC- and VN-tagged tau-WT or -P301L was similar in cells (Fig. 4B). We quantitatively measured the degree of tau aggregation by measuring the fluorescence generated after the complementary BiFC pair VN and VC was reconstituted back to the yellow fluorescent protein Venus. Our results showed that cells cotransfected with tau-P301L-VN and tau-P301L-VC pairs generated much stronger fluorescence than those cotransfected with tau-WT-VN and tau-WT-VC pairs, indicating a higher degree of tau oligomerization and/or aggregation in mutant tau-expressing cells (Fig. 4C). We demonstrated that induction of lysosome alkalinization by BafA1 can further enhance tau aggregation, while reacidification of lysosomal pH by tetrandrine treatment can reduce tau aggregation in a dose-dependent manner in mutant tau-expressing cells (Fig. 4C). This result suggests that tetrandrine can reduce tau aggregation, possibly by enhancing ALP function.

### Tetrandrine corrects impaired intracellular tau distribution and trafficking and promotes microglia-mediated tau clearance

**Fig. 5:**
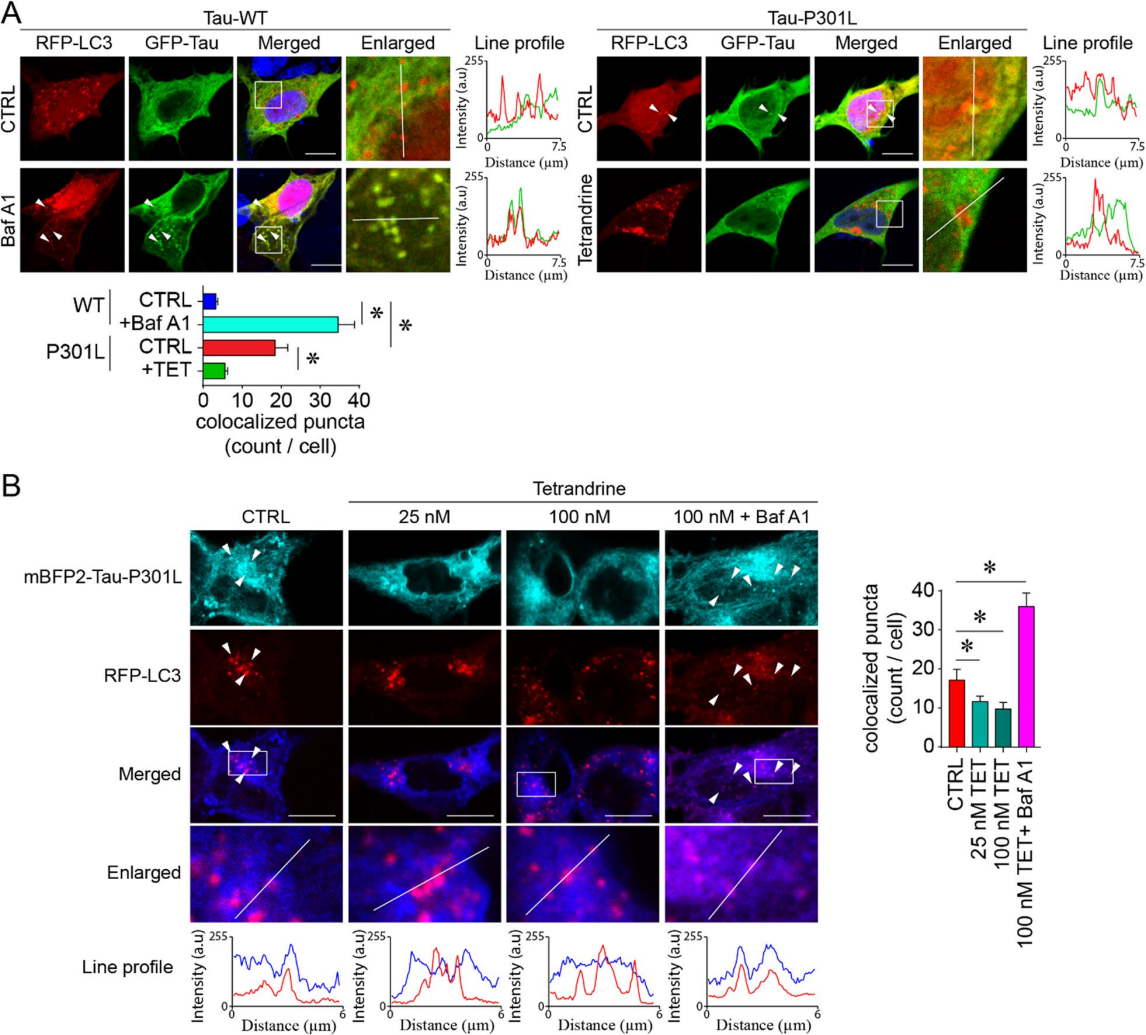
Tetrandrine promotes pathological tau degradation. **A** Representative micrographs showing autophagic degradation of tau tracked by GFP-Tau and RFP-LC3 in Tau-WT- or Tau-P301L-expressing SH-SY5Y cells. The enlarged images are the magnified regions indicated in the white boxes of the merged images. White arrowheads indicate the colocalization of tau and LC3 puncta, and their degree of colocalization was analysed by ImageJ with the colocalization analysis plugin. The red and green traces in the line profile show the intensity of red and green colour in arbitrary units (a.u.) along the white line indicated in the enlarged images. The bar chart depicts data summarized as the mean ± SEM from 3 individual experiments, with 30 cells counted in each group. *Indicates *p* < 0.05. **B** An analogous tau degradation experiment was performed using acid-resistant mBFP2-tagged tau P301L. The enlarged images are the magnified region indicated by the white boxes in the merged images. White arrowheads indicate the colocalization of tau-P301L—LC3 puncta under the influence of tetrandrine (TET) and bafilomycin A (BafA1). The degree of colocalization was analysed with ImageJ. The red and blue traces in the line profile show the intensity of red and blue colour in arbitrary units (a.u.) along the white line indicated in the enlarged images. The bar chart depicts data summarized as the mean ± SEM from 3 individual experiments, with 30 cells counted in each group. *Indicates *p* < 0.05

Tau aggregation is known to impair trafficking of organelles and vesicles [17]. These endosomal traffic jams can result from ALP impairment [42, 43]. By using mRFP-LC3 and GFP-tau to track ALP-mediated tau degradation, we found that the intracellular distribution of tau proteins in tau-P301L-expressing cells was different from that of the wild-type tau-expressing control cells (Fig. 5A). In contrast to the normal microtubule-like distribution of tau, tau-P301L tends to form puncta that colocalize with mRFP-LC3, suggesting the perturbation of ALP-mediated degradation of tau (Fig. 5A). We suspected that the occurrence of tau-P301L puncta is due to incomplete digestion in the alkalized LC3 autophagic vesicles. We recapitulated this pathogenic feature of tau-P301L aggregation in wild-type tau-expressing cells by treating the cells with BafA1, which causes lysosome alkalization (Fig. 5A). Intriguingly, the accumulation of tau-P301L puncta was markedly reduced by lysosome reacidification after tetrandrine treatment (Fig. 5A). As GFP-tau may be quenched by the acidic environment in lysosomes and autolysosomes, we performed an analogous experiment using acid-resistant mTagBFP2-tagged tau-P301L. Similar results were obtained, and tetrandrine rescued the impaired trafficking or degradation of tau-P301L-LC3, whereas the autophagy blocker BafA1 increased the abundance of these colocalized puncta (Fig. 5B). These results suggest that Tau-P301L increases TPC2 function and causes lysosome alkalization and impairment of autophagic degradation.

**Fig. 6:**
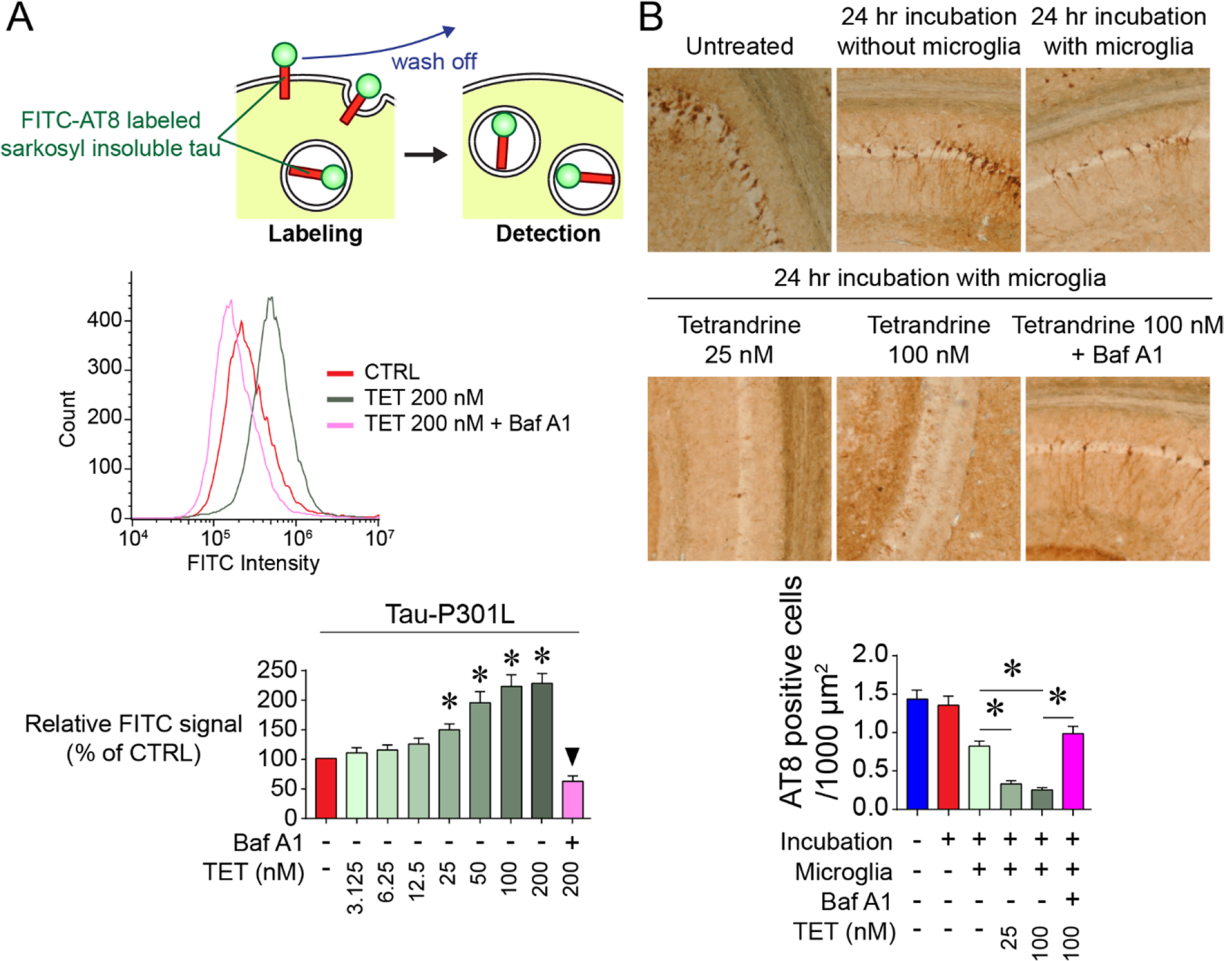
Tetrandrine promotes tau internalization and degradation by microglia. **A** Top, a schematic showing the internalization assay using FITC-AT8-labelled tau isolated from the Sarkosyl insoluble fraction of tau-P301S tau mouse brain homogenates. Middle, flow cytometry analysis depicting the internalization of FITC-AT8-labelled insoluble tau aggregates isolated from P301S tau mouse brain homogenates by microglia under the influence of TET and BafA1. Bottom, bar chart showing data summarized as the mean ± SEM from 5 individual experiments, *Indicates *p* < 0.05. ▼ indicates *p* < 0.05 compared to the 200 nM tetrandrine-treated group. **B** Representative micrographs showing the ex vivo tau-tangle clearance in 24 h in tau-P301S mouse brain slices under the influence of tetrandrine treatment at different concentrations. The bar chart depicts data summarized as the mean ± SEM from 5 individual experiments; *Indicates *p* < 0.05

To further investigate the trafficking and autophagic clearance of tau aggregates, we employed ex vivo approaches similar to those described by Luo et al. [35]. In brief, insoluble tau aggregates were isolated from the Sarkosyl-insoluble fraction of tau-P301S mouse brain homogenates. These insoluble tau aggregates were prelabelled with an FITC-AT8 antibody and incubated with microglial cultures. The uptake and trafficking of FITC-AT8 by microglia were then monitored by flow cytometry. Our results showed that tetrandrine promoted the internalization of FITC-AT8-labelled tau aggregates in a dose-dependent manner, whereas the autophagy inhibitor BafA1 prevented this process (Fig. 6A). To further verify the influence of tetrandrine on microglia-mediated tau clearance in the brain, ex vivo experiments were performed as described previously [35]. When brain slices from tau-P301S mice were incubated with microglia for 24 h, we observed a reduction in AT8-positive tau immunostaining compared to that in the untreated control slices and slices that were not incubated with microglia (Fig. 6B). Intriguingly, AT8 staining was further reduced when we treated the brain slices with microglia together with tetrandrine, suggesting that tetrandrine promotes microglia-mediated tau clearance. In contrast, cotreatment with tetrandrine and BafA1 significantly inhibited microglia-mediated tau clearance (Fig. 6B).

### Tetrandrine restores mutant tau-mediated microglial phagocytosis impairment and prevents pathological tau spreading

**Fig. 7:**
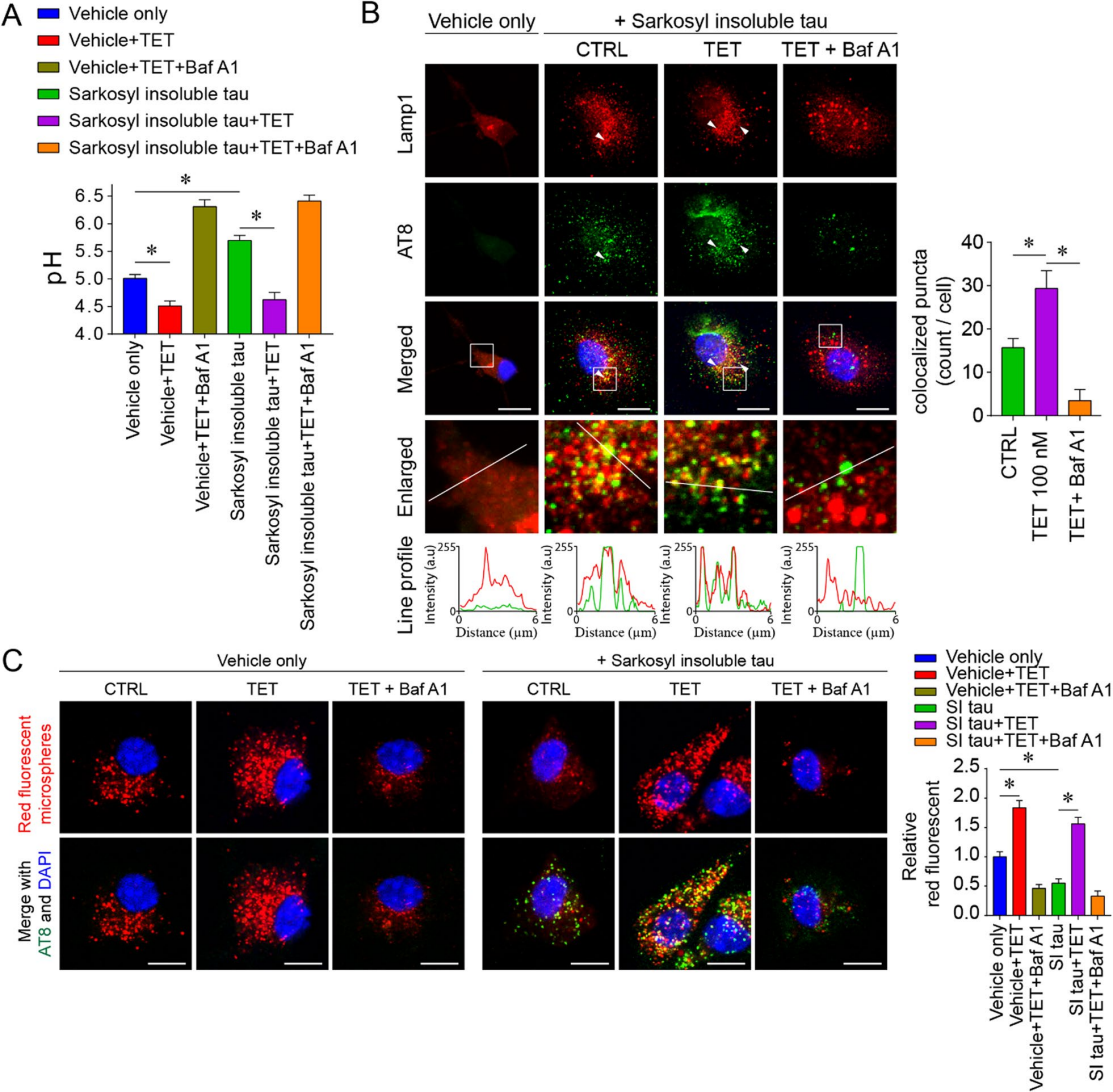
Tetrandrine restores the microglial phagocytosis impairment induced by pathological tau. **A** Lysosomal pH measurements in primary microglia 24 h after incubation with Sarkosyl-insoluble fractions from tau-P301S mouse brain homogenates with or without tetrandrine treatment. The Sarkosyl vehicle was used as the control. BafA1 was used to induce lysosomal alkalinization. Data are summarized as the mean ± SEM from 3 independent experiments. *Indicates *p* < 0.05. **B** Representative micrographs showing the localization of phagocytosed tau in microglia. Lamp1 and AT-8 were employed to label lysosomes and phosphor-tau, respectively. The enlarged images show the magnified region in the white boxes from the merged images. White arrowheads indicate the colocalization of tau and Lamp1, and their degree of colocalization was analysed with ImageJ with the colocalization plugin. The red and green traces in the line profile show the intensity of red and green colour in arbitrary units (a.u.) along the white line indicated in the enlarged images. Data are summarized as the mean ± SEM from 15 images analysed in each group. *Indicates *p* < 0.05. **C** Representative micrographs showing the phagocytosis assay of red fluorescent microspheres under the influence of tetrandrine (TET) and bafilomycin A1 (BafA1) in primary microglia 24 h after incubation with Sarkosyl-insoluble (SI) fractions of P301S tau mouse brain homogenates or Sarkosyl vehicle only. Data are summarized as the mean ± SEM from 15 images analysed in each group. *Indicates *p* < 0.05

In addition regulating lysosome pH, TPC can also promote endocytic membrane fusion [44]. However, microglial endocytic function is unlikely to benefit from exaggerated TPC2 activity. As microglial phagocytosis function is very sensitive to lysosome alkalinization (can be impaired by as little as 0.2 pH elevation) [45, 46], TPC2 overactivity would largely impair endocytic function by alkalizing lysosomes. Our in vitro assay showed that the internalization of pathological tau could disrupt microglial lysosome pH (Fig. 7A). Treatment with tetrandrine reacidified microglial lysosomes (Fig. 7A). In addition, we found that treatment with tetrandrine not only increased pathological tau uptake (Fig. 6A) but also increased colocalization with lysosomes, indicating that more phagocytosed materials could enter the late stage of endocytosis (Fig. 7B). By using red fluorescent microspheres to assay general microglial phagocytosis function, we found that the phagocytotic function of microglia was largely impaired after engulfment of pathological tau, therefore preventing further uptake (Fig. 7C). This finding is consistent with the recent finding that microglia lose endocytic function after engulfing tau tangle-stained neurons [47]. Our results showed that tetrandrine treatment reacidified lysosomes and restored microglial uptake activity (Fig. 7C). These results suggested that a nanomolar dose of tetrandrine could effectively restore lysosome homeostasis and ultimately restore the endocytic function impaired by mutant tau.

**Fig. 8:**
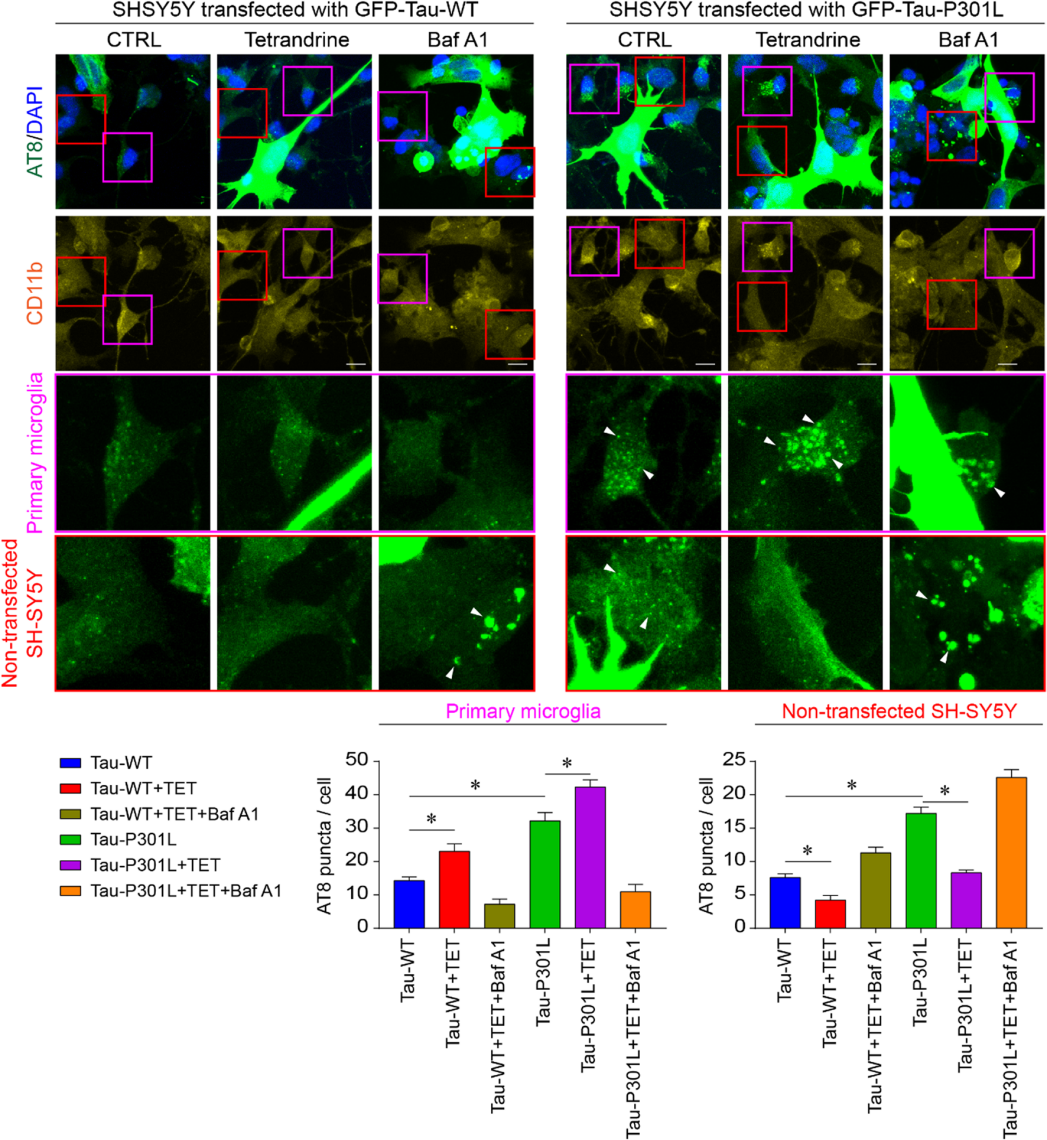
Tetrandrine reduces pathological tau transmission. Representative micrograph showing coculture of SH-SY5Y cells transfected with GFP-Tau-WT or GFP-tau-P301L with primary microglia. Hyperphosphorylated tau aggregates and primary microglial cells were stained with AT8 and CD11b, respectively. Red and purple boxes indicate the magnified regions for nontransfected SH-SY5Y cells and microglia, respectively. White arrowheads indicate transmission of AT8-positive puncta to primary microglia and nontransfected SH-SY5Y cells with or without tetrandrine or with BafA1. Bar charts depict the number of AT8-positive puncta transmitted to microglia or nontransfected SH-SY5Y cells. Data are summarized as the mean ± SEM from 15 images analysed in each group. *Indicates *p* < 0.05

Restoring microglial endocytic function could be vital to prevent tauopathy progression, as recent studies have shown that pathological tau may spread in a prion-like manner. This hyperphosphorylated tau may be secreted and taken up by adjacent cells [48]. To confirm this possibility, we transfected SH-SY5Y cells with GFP-tagged WT or mutant tau and cocultured them with primary microglia. We observed more AT8-positive puncta in primary microglia and untransfected SH-SY5Y cells in the GFP-Tau-P301L transfected culture, indicating the spreading of pathological tau to neighbouring cells. We also found that microglia could take up and clear these pathological tau secretions and prevent their spread (Fig. 8). Microglia-mediated tau clearance largely relies on lysosomal acidity, as BafA1 treatment reduced the number of AT8 puncta in microglia but increased the number of AT8 puncta in nontransfected SH-SY5Y cells. Treatment with tetrandrine promoted microglial uptake; thus, we observed fewer AT8 puncta in nontransfected SH-SY5Y cells (Fig. 8).

Taken together, our results indicate that pathological tau protein exaggerates TPC2 activity, which contributes to ALP impairment and leads to tauopathies. Targeting lysosomal TPC2 with tetrandrine restored lysosomal homeostasis and rescued ALP impairment, thereby mitigating disease progression.

## Discussion

Neurofibrillary tangle formation caused by hyperphosphorylated tau protein is one of the pathological hallmarks of Alzheimer’s disease (AD), frontotemporal dementia, and many other neurodegenerative diseases. Soluble neurotoxic tau oligomers can be degraded by catabolic mechanisms such as the ubiquitin‒proteasome system (UPS) and autophagy [49]. However, when hyperphosphorylated tau is deposited, it aggregates such that degradation through the UPS can no longer function efficiently. It is believed that insoluble aggregates cannot enter the proteasome barrel in the UPS [50]. Thus, the autophagy-lysosomal pathway (ALP) plays an indispensable role in the clearance of pathological tau aggregates. Whereas both the UPS and ALP are essential for the clearance of hyperphosphorylated tau, some studies have demonstrated that disrupting the ALP can exacerbate tau pathologies [51], suggesting that ALP is a more critical pathway for pathological tau clearance than the UPS [52].

In this study, we observed that lysosomal TPC2 Ca^2+^ signals are disrupted and exaggerated by pathological tau protein, causing an increase in lysosomal pH and ALP impairment (Figs. 3 and 4). Importantly, pathological tau protein has been shown to disrupt intracellular Ca^2+^ signalling [53, 54]. A recent proteomic study using two-month-old tau-P301S mice showed that multiple Ca^2+^ signalling pathways were altered during the early stage of tau pathogenesis [55]. Furthermore, another study on a Drosophila model showed that tau-R406W, a mutant tau that is more prone to hyperphosphorylation, can disrupt transcription by altering nuclear Ca^2+^ signalling via cyclic AMP (cAMP) response element-binding protein (CREB) [53]. Although TPC orthologues are not present in Drosophila, a study showed that the levels of CG42638 (predicted to be responsible for NAADP-sensitive Ca^2+^ release) and CG1943 (the human TPC2-associated protein orthologue JPT2 [56]) were elevated [53], suggesting that pathological tau may play a significant role in regulating NAADP-mediated Ca^2+^ signalling. It is not known whether a similar mechanism also contributes to the Ca^2+^ disruption observed in tau-P301S mice. Nonetheless, our quantitative qPCR assay showed that the expression of both TPC1 and TPC2, as well as CREB3 and CREB5, was increased in the hippocampus of Thy1-hTau.P301S mice (Fig S3A). In Western blot analysis, we observed an increase in total CREB levels but a decrease in phosphorylation levels (Fig S3B). This is consistent with the finding that the expression of the TPC gene is regulated by the cAMP/PKA/CREB signalling pathway [57]. Therefore, the observation of enhanced Ca^2+^ release from lysosomes in tau-P301L-expressing SH-SY5Y cells (Fig. 3A, B) may be attributed to an increase in the expression of TPC channels. Furthermore, disrupted lysosomal Ca^2+^ homeostasis could also be a consequence of the direct interaction of lysosomal membranes with tau aggregates. Studies have demonstrated that excessive α-synuclein oligomers in membranes disrupt membrane conductance and cause abnormal Ca^2+^ influx; similar observations have been made for tau and Aβ oligomers [58–60]. It is plausible that pathological tau can exacerbate TPC2-mediated lysosomal Ca^2+^ release via similar mechanisms. As lysosomal Ca^2+^ release increases, the lysosomal Ca^2+^ content is reduced (Fig. 3A, B). This reduction drives the expulsion of H^+^ from lysosomes through the Ca^2+^/H^+^ exchanger, thereby leading to lysosomal alkalinization [14, 61–63]. The increased pH in lysosomes impairs ALP function, as degradative enzymes such as cathepsin D require an acidic environment [64]. Therefore, lysosomal alkalinization will compromise ALP activity and increase pathological tau [51], exacerbating tau pathologies [65].

It is believed that pathological tau is liberated from cells via direct secretion of tau clusters, ectosomes, or exosomes or via junctions with adjacent cells [66]. These escaped tau molecules can be taken up by other cells in the brain, spreading tau pathologies [67]. Recent studies have shown that tau seeding and transmission play an important role in tauopathies [68, 69]. Those studies showed that tau seeds were unleashed into the cytosol when lysosomes were ruptured, and the spreading of tau could be mitigated by inhibiting lysosomal activity. Although it appears that hyperphosphorylated tau deposits are formed around acidic endocytic compartments, it is not certain whether lysosome acidity is the cause of release of engulfed pathogenic tau. ALP impairment is widely observed in AD and other neurodegenerative diseases [3, 70]. Some studies have suggested that lysosome alkalinization may cause rupture of lysosomes and lead to premature release of undigested content [71]. Therefore, it is believed that alkaline lysosomes are more harmful than acidic lysosomes. Our results show that pathological tau can cause excessive lysosomal Ca^2+^ release and increase the lysosomal pH, thereby contributing to the rupture of lysosomes and impairing the ALP-mediated clearance of tau.

Lysosome reacidification by tetrandrine has potential therapeutic value for tauopathies, as lysosomes must be acidified for their hydrolytic enzymes to function in degrading aged organelles and misfolded proteins during autophagy [72]. Accumulating evidence suggests that Aβ and tau are greatly influenced by neuronal autophagy and that their efficient degradation may prevent the pathogenesis and progression of AD [7, 73]. In addition, studies have shown that lysosomal acidity is reduced with ageing [74], thus creating a “suboptimal” environment that greatly impairs the activities of degradative enzymes required for autophagic clearance of Aβ and tau aggregates [51]. Pathological tau aggregates accumulate in autophagic vesicles in tau-P301L-expressing cells, indicating that their clearance by the ALP is impaired. Our results showed that tetrandrine mitigated lysosome alkalinization and thereby restored tau ALP clearance (Figs. 4 and 5). It has been shown that microglia can engulf extracellular tau and prevent tau seeds from spreading [75]. However, overactivity of TPC caused by pathological tau may interrupt endocytic activity [44]. In addition, overactivity of TPC also impairs the ALP in microglia such that tau taken up by microglia cannot be effectively cleared (Fig. 6). Nonetheless, it has been shown that microglia play important roles in the clearance of tau when their uptake function is enhanced [35]. Endocytic activity and subsequent endolysosome maturation require acidification, and lysosome alkalinization could disrupt the maturation of early endosomes [76, 77]. In this study, we found that tetrandrine-mediated rescue of lysosome pH can mitigate ALP and endocytic impairment that promoting pathological tau clearance (Fig. 6). However, when microglia engulf pathogenic tau aggregates, their phagocytotic function is lost [47]. The underlying reason why microglia become hypofunctional is not clear. However, in our study, we found that microglial lysosomes become more alkaline after engulfing insoluble tau aggregates isolated from tau-P301S mice (Fig. 7). Furthermore, we found that the impaired phagocytotic function observed in microglia after they engulfed insoluble tau aggregates could be reversed with tetrandrine treatment. These findings suggested that pathological tau could impair microglial phagocytotic function by lysosome alkalinization. As microglial phagocytosis is very sensitive to lysosome alkalinization [45, 46], pathological tau may promote tau seeding and transmission in a feed-forward manner. In our study, we observed more spreading of phosphor tau aggregates into nontransfected SH-SY5Y cells when the cells were treated with BafA1 or expressed mutant tau (Fig. 8). Treating the cells with tetrandrine promoted phosphor tau uptake in microglia and reduced transmission to nontransfected SH-SY5Y cells (Fig. 8).

In summary, our data suggest that hyperphosphorylated tau leads to TPC2 overactivity and impairs ALP function in neurons via lysosome alkalinization. Impairment of ALP-mediated clearance of tau may lead to premature release of undigested content and promote tau seeding and transmission [66, 71]. Microglia can cease such transmission by scavenging these aggregates [35, 78]. However, microglia are not fully functional due to lysosome alkalinization after phosphor tau engulfment. Reacidifying lysosomes with tetrandrine can restore ALP function to reduce pathological tau liberation from neurons and enhance the scavenging function of microglia to reduce pathological tau transmission. As autophagic impairment is increasingly recognized as an important hallmark of AD [7], it is believed that restoring lysosomal-mediated degradation via reacidification could be a therapeutic approach for many neurodegenerative diseases, such as Parkinson’s disease and AD [79].

## Conclusions

In conclusion, this study has shown that tetrandrine ameliorates tauopathies. By inhibiting TPC2 overactivity caused by pathological tau, tetrandrine restored ALP impairment and thus reduced tau deposition and mitigated tau-associated pathologies. In addition, tetrandrine enhanced microglial clearance of tau NFTs, leading to reduced neuroinflammation and improved memory functions in a dose-dependent manner. Reacidifying lysosomes with tetrandrine could be a therapeutic approach for tauopathy intervention.

## Supplementary Information

**Additional file 1**. Tetrandrine ameliorates cognitive deficits and mitigates tau aggregation in cell and animal models of tauopathies.

## Data Availability

All data needed to evaluate the conclusions in the paper are presented in the paper and/or the Supplementary Materials.

## Abbreviations

Aβ: Amyloid β
aCSF: Artificial cerebrospinal fluid
AD: Alzheimer’s disease
ALP: Autophagy-lysosomal pathway
a.u.: Arbitrary unit
BafA1: Bafilomycin A1
BiFC: Bimolecular fluorescence complementation
C57: C57BL6/J
cAMP: Cyclic adenosine monophosphate
CAX: Ca^2+^/H^+^ exchanger
CatD: Cathepsin D
CFC: Contextual fear conditioning
CREB: Cyclic adenosine monophosphate response element-binding protein
DIV: Days in vitro
fEPSP: Field excitatory postsynaptic potential
GFAP: Glial fibrillary acidic protein
GPN: Glycyl-L-phenylalanine-beta-naphthylamide
Iba-1: Ionized calcium binding adaptor molecule 1
i.p.: Intraperitoneal
LC3: Microtubule-associated protein 1A/1B-light chain 3
LTP: Hippocampal long-term potentiation
NCX: Na^+^/Ca^2+^ exchanger
NFT: Neurofibrillary tangles
NHE: Na^+^/H^+^ exchanger
NOR: Novel object recognition
SS: Sarkosyl-soluble
SI: Sarkosyl-insoluble
TET: Tetrandrine
TPC: Two-pore channel
UPS: Ubiquitin‒proteasome system
