# Supplementary material for: Tetrandrine ameliorates cognitive deficits and mitigates tau aggregation in cell and animal models of tauopathies
Source: J Biomed Sci. 2022 Oct 22;29:85. doi: 10.1186/s12929-022-00871-6 (PMC9587578; doi:10.1186/s12929-022-00871-6)
Supplement: Supplementary file 1 — Additional file 1. Tetrandrine ameliorates cognitive deficits and mitigates tau aggregation in cell and animal models of tauopathies. [file 12929_2022_871_MOESM1_ESM.docx]

**
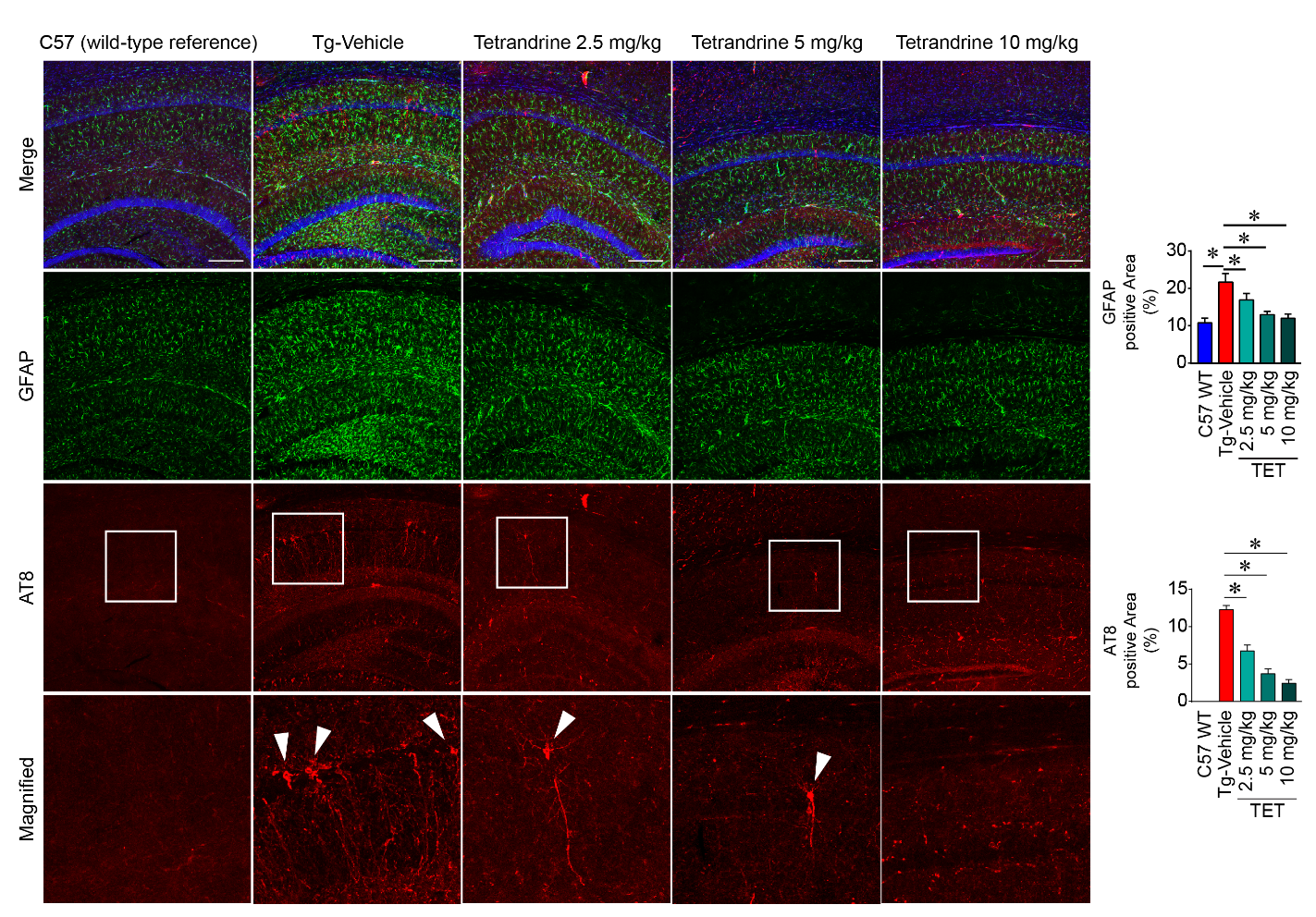
**

## Fig S1. Tetrandrine reduces GFAP (astrocytosis) and AT8 (phosphorylated tau) signals in Thy1-hTau.P301S mice

Micrographs showing representative immunofluorescence staining of astrocytosis and phosphor-tau in the hippocampal region in control C57 and Thy1-hTau.P301S mice treated with saline (vehicle) or different concentrations of tetrandrine (TET; 2.5 mg/kg, 5 mg/kg, and 10 mg/kg, ip injections every two days from 2 months old until 4 months old). After the treatment, brain slides were immuno-probed with GFAP and AT8 antibodies to reveal astrocytosis and hyperphosphorylated tau. The magnified images are enlarged regions of the white boxes indicated in the AT8 panel. The bar charts depict quantifications of GFAP and AT8 levels in mice. Data are summarized as the mean ± SEM from 8 mice, with 24 images analysed in each group. * indicates *p* < 0.05 compared with control C57 or vehicle-treated tau mice (Tg-Vehicle).


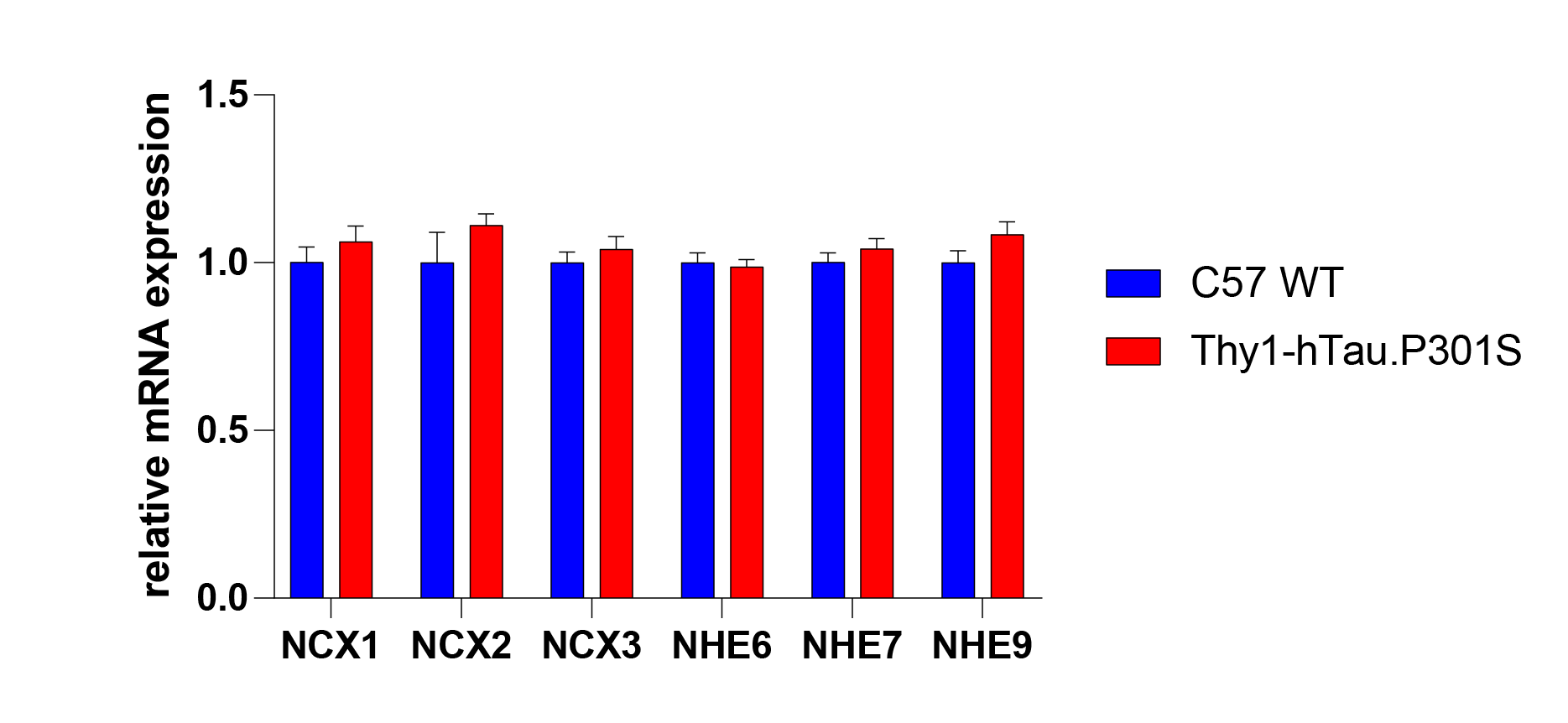


## Fig S2. Pathological tau does not alter the mRNA levels of sodium calcium exchanger (NCX) and sodium hydrogen exchanger (NHE)

Quantitative qPCR measurement of NCX1, NCX2, NCX3, NHE6 (localized in early recycling endosomes), NHE7 (localized in the trans-Golgi network) and NHE9 (localized in late recycling endosomes) mRNA in the hippocampus of C57 WT or Thy1-hTau.P301S mice. The bar chart depicts the relative mRNA expression of genes normalized to beta-actin. N = 8 for each group. Data are summarized as the mean ± SEM. No significant difference was found among the groups.


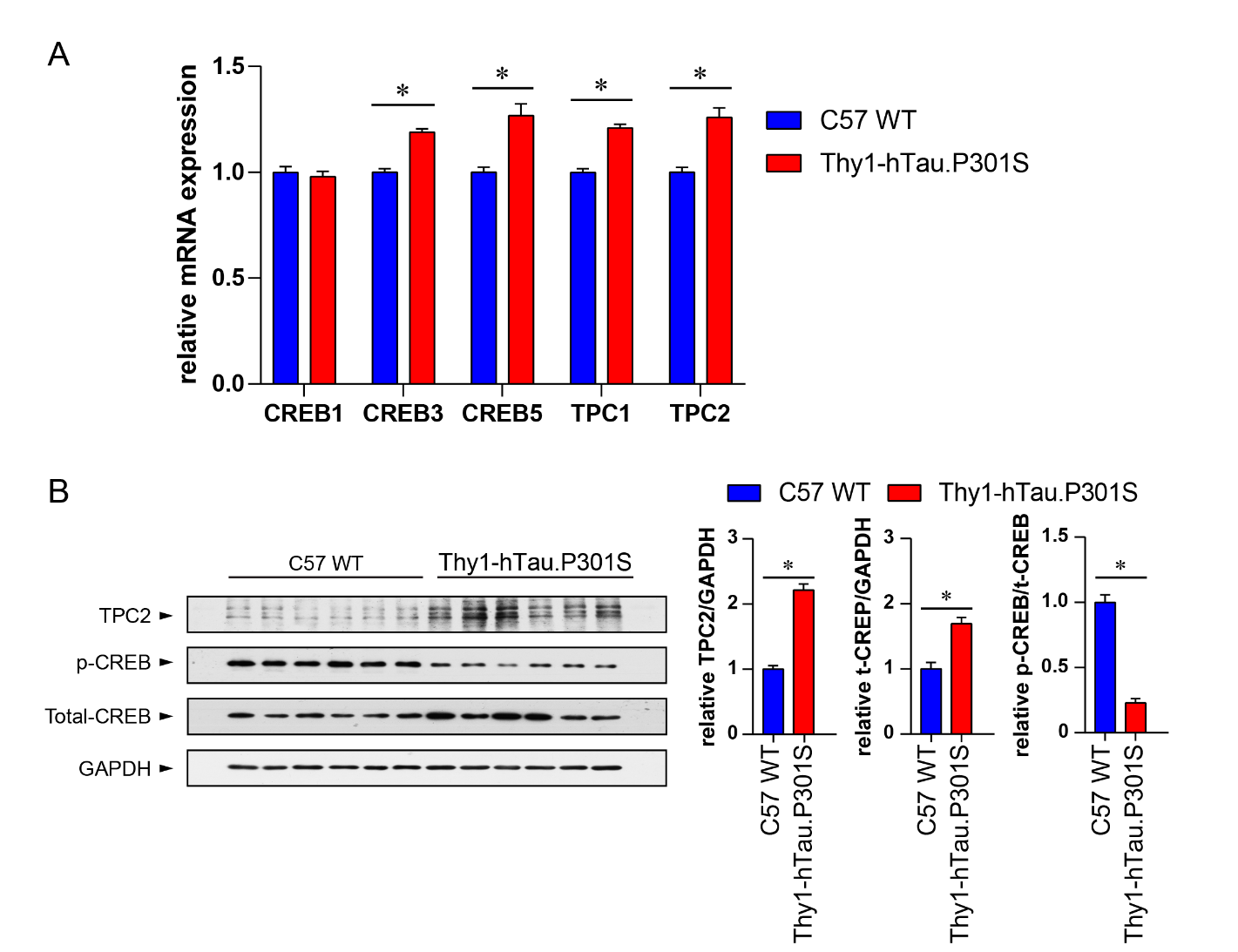


## Fig S3. Pathological tau disrupts TPC function *via* cyclic AMP (cAMP) response element-binding protein (CREB)

(A) Quantitative qPCR measurements of CREB1, CREB2, CREB3, TPC1 and TPC2 mRNA levels in the hippocampus of C57 WT or Thy1-hTau.P301S mice. The bar chart depicts the relative mRNA expression of genes normalized to beta-actin. N = 8 for each group. * indicates *p* < 0.05 compared to C57 WT mice. (B) Representative Western blots showing the protein expression of TPC2, total CREB and p-CREB in brain lysates from C57 WT or Thy1-hTau.P301S mice. The bar charts on the right summarize the protein levels in the mouse hippocampus normalized to GAPDH. Data are summarized as the mean ± SEM. N = 8 for each group. * indicates *p* < 0.05 compared to the expression of C57 WT.
